# Adherence to Antiretroviral Prophylaxis for HIV Prevention: A Substudy Cohort within a Clinical Trial of Serodiscordant Couples in East Africa

**DOI:** 10.1371/journal.pmed.1001511

**Published:** 2013-09-10

**Authors:** Jessica E. Haberer, Jared M. Baeten, James Campbell, Jonathan Wangisi, Elly Katabira, Allan Ronald, Elioda Tumwesigye, Christina Psaros, Steven A. Safren, Norma C. Ware, Katherine K. Thomas, Deborah Donnell, Meighan Krows, Lara Kidoguchi, Connie Celum, David R. Bangsberg

**Affiliations:** 1 Center for Global Health, Massachusetts General Hospital, Boston, Massachusetts, United States of America; 2 Department of Medicine, Harvard Medical School, Boston, Massachusetts, United States of America; 3 Department of Global Health, University of Washington, Seattle, Washington, United States of America; 4 Department of Medicine, University of Washington, Seattle, Washington, United States of America; 5 Department of Epidemiology, University of Washington, Seattle, Washington, United States of America; 6 US Centers for Disease Control and Prevention, Entebbe, Uganda; 7 Infectious Disease Institute, Makerere University, Kampala, Uganda; 8 University of Manitoba, Department of Infectious Diseases, Winnipeg, Canada; 9 Kabwohe Clinical Research Center, Kabwohe, Uganda; 10 Department of Psychiatry and Behavioral Medicine, Massachusetts General Hospital, Boston, Massachusetts, United States of America; 11 Department of Psychiatry, Harvard Medical School, Boston, Massachusetts, United States of America; 12 Department of Global Health and Social Medicine, Harvard Medical School, Boston, Massachusetts, United States of America; 13 Fred Hutchinson Cancer Research Center, Seattle, Washington, United States of America; 14 Department of Medicine, Mbarara University of Science and Technology, Mbarara, Uganda; Medical Research Council of South Africa, South Africa

## Abstract

**Background:**

Randomized clinical trials of oral antiretroviral pre-exposure prophylaxis (PrEP) for HIV prevention have widely divergent efficacy estimates, ranging from 0% to 75%. These discrepancies are likely due to differences in adherence. To our knowledge, no studies to date have examined the impact of improving adherence through monitoring and/or intervention, which may increase PrEP efficacy, or reported on objective behavioral measures of adherence, which can inform PrEP effectiveness and implementation.

**Methods and Findings:**

Within the Partners PrEP Study (a randomized placebo-controlled trial of oral tenofovir and emtricitabine/tenofovir among HIV-uninfected members of serodiscordant couples in Kenya and Uganda), we collected objective measures of PrEP adherence using unannounced home-based pill counts and electronic pill bottle monitoring. Participants received individual and couples-based adherence counseling at PrEP initiation and throughout the study; counseling was intensified if unannounced pill count adherence fell to <80%. Participants were followed monthly to provide study medication, adherence counseling, and HIV testing. A total of 1,147 HIV-uninfected participants were enrolled: 53% were male, median age was 34 years, and median partnership duration was 8.5 years. Fourteen HIV infections occurred among adherence study participants—all of whom were assigned to placebo (PrEP efficacy = 100%, 95% confidence interval 83.7%–100%, *p*<0.001). Median adherence was 99.1% (interquartile range [IQR] 96.9%–100%) by unannounced pill counts and 97.2% (90.6%–100%) by electronic monitoring over 807 person-years. Report of no sex or sex with another person besides the study partner, younger age, and heavy alcohol use were associated with <80% adherence; the first 6 months of PrEP use and polygamous marriage were associated with >80% adherence. Study limitations include potential shortcomings of the adherence measures and use of a convenience sample within the substudy cohort.

**Conclusions:**

The high PrEP adherence achieved in the setting of active adherence monitoring and counseling support was associated with a high degree of protection from HIV acquisition by the HIV-uninfected partner in heterosexual serodiscordant couples. Low PrEP adherence was associated with sexual behavior, alcohol use, younger age, and length of PrEP use.

*Please see later in the article for the Editors' Summary*

## Abstract 2

Jessica Haberer and colleagues investigate the association between high adherence to antiretroviral pre-exposure prophylaxis and HIV transmission in a substudy of serodiscordant couples participating in a clinical trial.

*Please see later in the article for the Editors' Summary*

## Editors' Summary

**Background:**

Every year, about 2.5 million people (mostly living in sub-Saharan Africa) become infected with HIV, the virus that causes AIDS. HIV, which is usually transmitted through unprotected sex with an HIV-infected partner, destroys immune system cells, leaving infected individuals susceptible to other infections. There is no cure for AIDS, although antiretroviral drugs can hold HIV in check, and there is no vaccine against HIV infection. Individuals can reduce their risk of HIV infection by abstaining from sex, by having only one or a few low risk sexual partners, and by always using a condom. In addition, antiretroviral drugs can potentially be used in two ways to reduce HIV transmission. First, these drugs could be given to HIV-positive individuals to reduce their infectiousness. Second, antiretroviral drugs could be given to HIV-uninfected people to reduce acquisition of the virus. This approach—pre-exposure prophylaxis (PrEP)—has provided varying levels of protection against HIV infection in randomized controlled trials (RCT; studies that monitor the outcomes of groups of patients randomly assigned to receive different test drugs or a placebo/dummy drug).

**Why Was This Study Done?:**

One hypothesis for the varying efficacy of PrEP in RCTs is differential adherence—differences in whether trial participants took the antiretroviral drugs correctly. Antiretroviral drugs only control HIV infections effectively when they are taken regularly and adherence to antiretroviral PrEP is probably also important for HIV prevention. Here, the researchers investigate adherence to antiretroviral prophylaxis in a substudy within the Partners PrEP Study, a placebo-controlled RCT of oral antiretroviral drugs among nearly 5,000 HIV-uninfected members of serodiscordant couples in East Africa. In serodiscordant couples, only one partner is HIV-positive; 20% of couples in Africa who know their HIV status are serodiscordant. In the Partner PrEP Study, the efficacy of HIV protection with oral antiretroviral drugs was 67%–75%.

**What Did the Researchers Do and Find?:**

The researchers selected a “convenience” sample—a sample is taken non-randomly from a population that is close at hand—of 1,147 HIV-uninfected partners enrolled in Uganda. They used unannounced home-based pill counts (an approach that reduced the chance of participants dumping unused pills to appear more adherent than they actually were) and electronic pill bottle monitoring (a microchip in the medication bottle cap recorded whenever the bottle was opened) to measure PrEP adherence in this cohort. All the participants received adherence counseling at PrEP initiation and throughout the study; counseling was intensified if unannounced pill count adherence fell below 80%. Fourteen participants, all of whom had been assigned to placebo, became HIV-positive during the adherence substudy. The average adherence to PrEP was 99.1% and 97.2% as measured by unannounced pill counts and by electronic monitoring, respectively. About 7% and 26% of participants had less than 80% adherence as measured by unannounced pill count and electronic monitoring, respectively, during at least one 3-month period of the substudy. Greater than 80% adherence was associated with the first 6 months of PrEP use and polygamous marriage. Adherence less than 80% was associated with report of no sex or sex with another person besides the study partner, younger age, and heavy alcohol use. Finally, the adherence intervention (intensified counseling) was well received and in the first unannounced pill count after the intervention, adherence increased to above 80% in 92% of participants.

**What Do These Findings Mean?:**

These findings indicate that the high level of PrEP adherence achieved in the setting of active adherence monitoring and counseling support was associated with a high level of protection from HIV acquisition by the HIV-uninfected partner in heterosexual serodiscordant couples. The findings also suggest that low PrEP adherence is associated with sexual behavior, alcohol use, younger age, and length of PrEP use. Several aspects of the study design may limit the accuracy of these findings. For example, although the adherence measures used here are probably more accurate than participant reports of missed doses and clinic-based pill counts (adherence measures that are often used in RCTs), they are not perfect. Nevertheless, these findings provide further support for the ability of PrEP to prevent HIV acquisition when taken regularly; they suggest that adherence interventions in the implementation setting should address sexual behavior, risk perception, and heavy alcohol use; and they provide data to guide ethical decisions about resource allocation for prevention and treatment of HIV infection.

**Additional Information:**

Please access these Web sites via the online version of this summary at http://dx.doi.org/10.1371/journal.pmed.1001511.

- The 2012 UNAIDS World AIDS Day Report provides up-to-date information about the AIDS epidemic and efforts to halt it
- Information is available from the US National Institute of Allergy and infectious diseases on HIV infection and AIDS
- NAM/aidsmap provides basic information about HIV/AIDS, summaries of recent research findings on HIV care and treatment, and information on HIV transmission and prevention and on PrEP
- Information is available from Avert, an international AIDS charity, on many aspects of HIV/AIDS, including information on HIV and AIDS in Uganda, on HIV prevention, and on PrEP (in English and Spanish)
- PrEP Watch provides detailed information about PrEP and links to other resources; it includes personal stories from people who have chosen to use PrEP
- More information about the Partners PrEP Study is available
- Personal stories about living with HIV/AIDS are available through Avert, through Nam/aidsmap, and through the charity website Healthtalkonline

## Introduction

Over 2.5 million people are infected with HIV each year globally [1]. HIV antiretroviral medications, whether given to an HIV-infected person to reduce infectiousness or as pre-exposure prophylaxis (PrEP) to an HIV-uninfected person to prevent acquisition, hold great promise for decreasing the number of new infections. PrEP has strong biologic plausibility for HIV prevention [2]; however, randomized clinical trials of PrEP have generated conflicting results. Three studies have shown protection against HIV infection with efficacy estimates ranging from 44%–75% [3]–[5], while two other studies have been stopped in whole or in part because of futility to demonstrate efficacy [6],[7].

Adherence to antiretroviral medications is essential for efficacious treatment of HIV infection [8], and adherence to antiretroviral PrEP is also likely important for HIV prevention. Thus, differential adherence across clinical trials of PrEP is the leading hypothesis to explain the differences in clinical trial efficacy estimates [9],[10]. Supporting this theory, trials demonstrating efficacy for HIV protection have shown close relationships between detection of antiretroviral medications in blood samples and HIV protection [3],[4]. Notably, two of the trials that failed to demonstrate PrEP efficacy detected antiretroviral medication in blood samples from only a minority of participants [7],[11]. Moreover, a recent modeling study indicated 99% risk reduction of HIV infection when PrEP is taken 7 days a week [12].

Clinical trials of PrEP have used several measures to estimate adherence to the study medication, including participant reports of missed doses, clinic-based pill counts of unused medication, and blood levels of the antiretroviral medications. Each measure has important limitations. Participant report often overestimates adherence owing to social desirability bias and failure to remember missed doses [13]. Clinic-based pill counts are an objective measure; however, they are often susceptible to participant manipulation prior to the clinic visit (i.e., pill dumping) [14]. Blood levels of antiretroviral medications are similarly subject to manipulation in that participants may take medications just before a scheduled study visit when they know that drug levels will be drawn [15]. Moreover, because drug levels are subject to both behavioral (i.e., time of dosing) and biological variation (i.e., pharmacokinetics), they may poorly correlate with actual adherence behavior; in one study of antiretroviral treatment, blood levels of drug were only modestly associated with HIV viral suppression [16]. Objective behavioral adherence measures may improve understanding of the relationship between adherence behavior and PrEP protection against HIV. Additionally, other trials have not systematically involved the provision of further adherence support for those with poor adherence who would like to continue taking PrEP in the trial.

Within a randomized, placebo-controlled, clinical trial of daily oral PrEP (the Partners PrEP Study), we enrolled subjects into a substudy designed to monitor and improve adherence. Two objective measures of adherence behavior (unannounced home-based pill counts [UPC] and the medication event monitoring system [MEMS]), were utilized to monitor adherence. A two-stepped approach to adherence counseling was also employed, which involved initial adherence counseling, followed by more intensive counseling for those who fell to <80% adherence from the UPC monitoring. Here, we estimate the efficacy of PrEP in the context of both intensive adherence monitoring and counseling, as well as characterize PrEP adherence behavior and examine factors associated with low adherence.

## Methods

### Ethics Statement

The study protocol was approved by the human subjects committees of Massachusetts General Hospital/Partners Healthcare, the University of Washington, the Centers for Disease Control and Prevention, the Uganda National Council for Science and Technology, and the Uganda Virus Research Institute Science and Ethics Committee.

### Partners PrEP Study

The Partners PrEP Study was a phase III, randomized, double-blind, placebo-controlled, three-arm clinical trial of daily oral tenofovir (TDF) and emtricitabine/tenofovir (FTC/TDF) PrEP provided to HIV-uninfected members of 4,758 HIV serodiscordant couples attending nine clinical research sites in Kenya and Uganda. Enrollment began in July 2008 and concluded in November 2010. Retention was high at 97% at 1 year and 96% at 2 years of individual follow-up. The design, procedures, and outcomes of the Partners PrEP Study clinical trial are described elsewhere [3]. Briefly, HIV-uninfected partners were randomly assigned to once-daily TDF, combination FTC/TDF, or matching placebo and followed monthly for safety assessments and HIV seroconversion for up to 36 mo. Adherence was measured with clinic-based pill counts and self-report at the monthly visits. HIV-infected partners were not eligible for antiretroviral therapy under national guidelines at the time of enrollment, but were monitored and actively referred for antiretroviral treatment initiation if they became eligible during the course of follow-up. All couples received a package of HIV prevention services, including risk-reduction counseling, couples counseling, and condoms. In July 2011, the independent Data and Safety Monitoring Board recommended public report of the results and discontinuation of the trial placebo arm due to demonstration of 67% efficacy for HIV protection with TDF and 75% efficacy with FTC/TDF.

### Adherence Substudy

In November 2009, we initiated a substudy to objectively measure and support adherence at three of the Partners PrEP Study sites (Kabwohe, Kampala, and Tororo: all in Uganda). A convenience sample was selected from those already enrolled or simultaneously enrolling in the main clinical trial and who had at least 6 mo of follow-up remaining in the main clinical trial; participants included all study arms (which were blinded at the time) and no other selection criteria were used. In the adherence substudy, additional adherence assessment was performed using two validated objective measures. First, UPC were conducted at the participant's home unannounced (i.e., participants were not informed of the date of the visit) on a randomly selected day every month for the first 6 mo and quarterly thereafter. The random nature of the visit was intended to reduce the chance that participants would manipulate pill bottles (i.e., dump pills) prior to the measurement. Second, MEMS (Aardex) were used to electronically record the date and time of pill bottle openings; data were downloaded monthly. Both UPC and MEMS have been closely correlated with each other and with HIV RNA suppression when measured in HIV-infected individuals on antiretroviral therapy in Uganda and San Francisco [17],[18], although both measures are still susceptible to manipulation. Participants found to have UPC adherence <80% were enrolled in a manualized, customizable, multi-session adherence intervention [19]. The intervention modules were consistent with principles of cognitive behavioral therapy and problem-solving therapy. Accordingly, the intervention began with psycho-educational information and rapport building, and later involved motivational interviewing and assistance with specific problem-solving strategies. Because the study population consisted of individuals in serodiscordant partnerships, the intervention included a couples-based component, such that the initial portion of the session was conducted with just the participant taking PrEP, and the second part with both members of the dyad (optional, but encouraged). The intervention was designed to be approximately 30–45 min long at the initial session with shorter subsequent sessions, and participants could have as many sessions as they or the counselors felt would be useful (average 6.8 per participant taking PrEP with range of 1 to 16). This article presents data collected through the July 2011 announcement of HIV protection efficacy in the main clinical trial, at which time enrollment in the substudy concluded.

### Statistical Analysis

All statistical analyses were conducted with SAS 9.2 and Stata 12.0. Characteristics of study participants enrolled and not enrolled in the adherence study were compared with Fisher's exact test for categorical covariates, and Wilcoxon rank sum test for continuous covariates. Efficacy of PrEP while in the adherence substudy was estimated by 1 minus the incidence rate ratio (IRR). The 95% exact confidence interval for the IRR was used.

Adherence by UPC and MEMS was estimated by the number of pills taken during the study quarter divided by the number of days the participant would be expected to take the pills, excluding days when a protocol-defined drug hold was in effect (e.g., for adverse events or pregnancy, which was defined by a positive urine test performed at each monthly visit in the Partners PrEP Study). Overall participant adherence was calculated using this same method, except that the interval in question was the entire study period for that individual rather than the quarter. When UPC was performed once a quarter (i.e., after 6 mo of follow-up), the UPC was used to estimate how many pills had been taken since the last clinic visit; clinic pill count data were used to estimate adherence during the time between visits. MEMS data were unadjusted except to account for pill bottle openings by study staff. Adherence values >100% may have occurred due to additional doses (e.g., multiple pills taken per day) or limitations of the adherence measurements. For instance, a participant may have manipulated the pill count (i.e., dumped pills prior to the measurement) or a participant may have opened a MEMS bottle numerous times without removing pills (e.g., due to curiosity). UPC and MEMS adherence were compared by Spearman's correlation. Low adherence was defined as <80% adherence in a quarter, paralleling the trigger used for the adherence intervention in this study. The threshold value of 80% was chosen based on biologic plausibility [20] and is consistent with high adherence as defined in another PrEP study [21], although the exact level of adherence needed to protect against HIV acquisition is unknown.

Potential associations with <80% UPC and MEMS adherence were evaluated using univariable and multivariable (adjusted) generalized estimating equation (GEE) models with logistic link and robust standard errors to account for repeated measures. Variables assessed on a monthly basis were categorized to reflect any reported behaviors during the quarter (e.g., no sex indicates no sex in the entire quarter). Variables were measured concurrently with adherence behavior. Enrollment and time-varying characteristics were assessed for both the HIV-uninfected and HIV-infected partners. Socio-economic status index was evaluated via a principal components analysis based on the Filmer-Pritchett Index and involved the presence of running water, a concrete floor, electricity, a metal roof, a television, and two or more rooms in the residence [22]. Heavy alcohol use was defined as a positive Rapid Alcohol Problems Screen [23]. Depression was assessed by the Hopkins Checklist, using 1.75 as a cut-off [24]. Belief in PrEP efficacy was assessed by standardized questionnaire prior to the release of efficacy data in July 2011. Adjustment in the multivariable model was for site and variables for which the *p*-value on univariable analysis was <0.10. Where CD4 count at enrollment and at follow-up were both significantly related at *p*<0.10, only the stronger CD4 count variable was carried forward to the multivariate analysis. The presence of different effects by gender for sex behaviors and for polygamous relationships were evaluated by testing interaction terms with gender in the GEE model; these variables were chosen a priori as likely to have different effects on adherence by gender.

## Findings

### Study participants

A total of 1,185 seronegative participants were considered for enrollment in the adherence substudy; 38 (3.2%) were not enrolled due to refusal, having less than 6 mo of follow-up remaining in the clinical trial, or logistical reasons that would interfere with home visits; 1,147 participants were enrolled in the study, reflecting 66% of all participants in the three study sites. Table 1 shows the individual and partnership characteristics for the participants in the Partners PrEP Study and in the adherence substudy (total and by arm in the substudy) at enrollment. Characteristics are also presented for those Partners PrEP Study participants who were based in the sites of the adherence substudy, but did not participate in the adherence substudy.

**Table 1:** Enrollment characteristics of study participants.

Characteristics	Partners PrEP Participants	Adherence Substudy Sites				
		Total	TDF Arm	FTC/TDF Arm	Placebo Arm	Participant Not Enrolled^a^
	*n* (%) or Median (IQR)					
***Individual characteristics***	*n* = 4,747	*n* = 1147	*n* = 359	*n* = 386	*n* = 402	*n* = 597
Male gender	2,962 (62%)	608 (53%)	196 (55%)	203 (53%)	209 (52%)	349 (58%)
Years of education	7 (4–10)	6 (3–7)	6 (3–8)	6 (3–8)	6 (3–8)	6 (3–9)
Age in years	33 (28–40)	34 (30–40)	34 (29–40)	35 (30–40)	34 (30–40)	34 (28–40)
Placebo	1,584 (33%)	402 (35%)	n/a	n/a	n/a	183 (31%)
Entry into the adherence study						
Concurrent with trial enrollment	n/a	290 (25%)	97 (27%)	100 (26%)	93 (23%)	n/a
Months 1–6	n/a	182 (16%)	62 (17%)	56 (15%)	64 (16%)	n/a
Months 7–12	n/a	202 (18%)	61 (17%)	68 (18%)	73 (18%)	n/a
After month 12	n/a	473 (41%)	139 (39%)	162 (42%)	172 (43%)	n/a
***Partnership characteristics***						
Married	4,635 (98%)	1,135 (99%)	353 (98%)	383 (99%)	399 (99%)	581 (97%)
Living together	4,650 (98%)	1,129 (98%)	353 (98%)	382 (99%)	394 (98%)	585 (98%)
Number of years living together	7.0 (3.0–14.0)	8.5 (3.7–15.3)	8.2 (3.6–15.0)	8.0 (3.7–15.3)	9.0 (3.8–15.9)	7.1 (3.0–14.2)
Number of children in the partnership	2 (1–4)	2 (1–4)	2 (1–4)	2 (1–4)	2 (1–4)	2 (1–4)
Polygamous marriage	974 (21%)	282 (25%)	82 (23%)	104 (27%)	96 (24%)	158 (27%)
Age difference between partners	1 (−4 to 6)	0 (−5 to 5)	1 (−6 to 5)	0 (−6 to 5)	0 (−5 to 5)	0 (−6 to 6)
Unprotected sex in prior month	1267 (28%)	321 (29%)	107 (30%)	111 (30%)	103 (26%)	142 (25%)
HIV-infected partner CD4 count (cells/mm^3^)	495 (375–662)	491 (368–667)	464 (348–626)	503 (380–682)	504 (372–687)	477 (355–645)
HIV-infected partner viral load (log copies/ml)	3.9 (3.2–4.5)	4.0 (3.3–4.6)	4.1 (3.4–4.6)	3.9 (3.2–4.5)	4.0 (3.4–4.6)	4.0 (3.3–4.6)

Complete data were available on all variables (*n* = 1,147) except for questions regarding unprotected sex in the prior month (missing in 3%), polygamy (<1%), and viral load (1%).

a From the three sites from which the adherence substudy recruited.

n/a, not applicable.

Among participants in the adherence substudy, 53% were male, the median age was 34 y (interquartile range [IQR] 30–40), and 35% were taking placebo. Nearly all (99%) were married with a median duration of partnership of 8.5 y (IQR 3.7–15.3) and 29% reported unprotected sex within the past month. The median CD4 count for the HIV-infected partner was 491 cells/µl (IQR 368–667). When comparing participants in the adherence substudy to participants in the overall Partners PrEP Study, notable differences include fewer males (53% versus 62%), somewhat longer partnerships (median 8.5 y versus 7.0 y), and a slightly higher rate of polygamy (25% versus 21%). These differences in male gender and partnership duration were also seen when comparing individuals who did and did not participate in the adherence substudy at the three sites where the substudy took place; however, rates of polygamous marriage were more similar (25% versus 27%). Additionally, more participants were on placebo (34% versus 31%) and unprotected sex in the prior month was somewhat more common (29% versus 25%). Characteristics across study arms were very similar.

Because most participants enrolled in the adherence substudy subsequent to their enrollment in the clinical trial, varying periods of time on PrEP were observed; specifically, 388 (34%) participants contributed data during 0–6 mo on PrEP, 593 (52%) during 7–12 mo, 606 (53%) during 13–18 mo, 540 (47%) during 19–24 mo, and 385 (34%) beyond 24 mo. Retention was high at 94% and 93% at 12 mo and 18 mo, respectively, for clinic visits, and 83 and 89% for 12 and 18 mo, respectively, for home visits. Average follow-up was 11.3 study mo (standard deviation [SD] 5.2).

### PrEP efficacy

Among participants enrolled in the adherence substudy, 14 acquired HIV during follow-up. All 14 were participants randomized to placebo (among 404 participants contributing 333 person-years). Participants randomized to the two active PrEP arms acquired 0 infections (among 750 participants contributing 616 person-years), indicating that PrEP efficacy for HIV prevention in the adherence substudy population was 100% (95% CI 83.7%–100%, *p*<0.001).

### Summary of adherence

**Figure 1:**
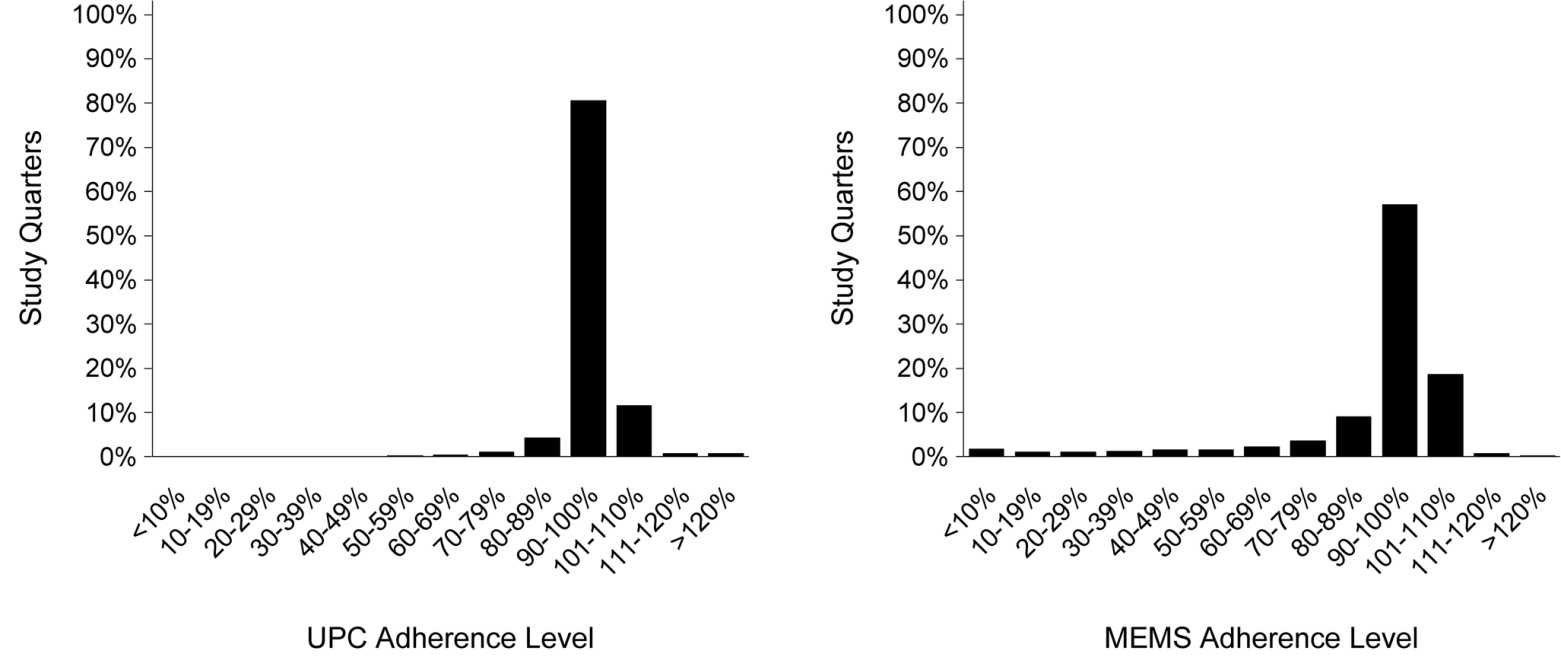
Distribution of adherence by unannounced pill count and electronic monitoring.

Objective behavioral adherence measures from the adherence substudy are summarized in Table 2. Median overall participant adherence was 99.1% (IQR 96.9%–100%) by UPC and 97.2% (IQR 90.6%–100%) by MEMS. Adherence was similar between genders, among the study arms, and over time. Single openings per day were recorded for 96.7% of all days covered in the study, and 95.0% of the remaining days indicated two openings. Because those openings may have reflected true dosing behavior (e.g., one pill early one morning and another pill late that night for use during the next day), MEMS data were not adjusted for analysis. As shown in Figure 1, the distribution of adherence includes values >100%, but adherence was >110% in only 1.5% of quarters measured by UPC and 1.0% of quarters measured by MEMS. UPC and MEMS were significantly correlated at 0.5 (*p*<0.0001). A total of 71 (6.8%) and 282 (25.8%) participants had <80% adherence for at least one quarter during the study by UPC and MEMS, respectively. Greater than 80% adherence was seen at 6 mo, 12 mo, 18 mo, and 24+ mo of PrEP use in 97.6%, 96.8%, 97.5%, and 98.7% of participants by UPC and 86.2%, 82.2%, 85.4%, and 87.8% by MEMS, respectively. Pill sharing was reported by no participants in the adherence substudy.

**Table 2:** Summary of adherence by measure.

Description	Unannounced Pill Count			MEMS		
	Median (IQR)	Mean (SD)	*n*	Median (IQR)	Mean (SD)	*n*
Overall	99.1% (96.9–100%)	97.6% (7.1)	1,041^a^	97.2% (90.6–100%)	91.1% (17.2)	1,093^a^
By site						
Kabwohe	99.1% (97.5–100%)	97.2% (7.7)	349	98.2% (92.9–100%)	95.1% (9.3)	357
Kampala	98.8% (96.1–100%)	97.0% (8.1)	351	92.9% (77.7–97.8%)	82.6% (23.2)	369
Tororo	99.4% (97.5–100%)	98.5% (5.1)	341	98.9% (94.8–100%)	95.9% (12.2)	367
By gender						
Female	99.3% (97.4–100%)	98.0% (6.6)	491	98.2% (92.9–100%)	93.7% (14)	509
Male	98.8% (96.3–100%)	97.3% (7.6)	550	96.2% (88.3–99.5%)	88.9% (19.3)	584
By study arm						
TDF	99.1% (96.5–100%)	97.0% (8.6)	352	96.9% (90.5–100%)	90.4% (18.4)	339
FTC/TDF	99.2% (97.2–100%)	97.8% (6.5)	367	97.3%(90.8–100%)	91.6%(16.8)	367
Placebo	99.1% (96.7–100%)	97.9% (6.4)	322	97.3% (90.5–100%)	91.4% (16.5)	387
By quarter since enrollment into the adherence substudy						
Q1 (M1–3)	100.0% (97.1–100%)	98.5% (11.8)	922	98.8% (92.9–100%)	93.8 (15.3)	1,093
Q2 (M4–6)	100.0% (97.1–100%)	98.2% (7.7)	933	97.6% (91.7–100%)	91.8 (18.6)	946
Q3 (M7–9)	100.0% (96.5–100%)	97.8% (8.3)	686	97.6% (91.5–100%)	89.5 (23.1)	799
Q4 (M10–12)	98.9% (96.2–100%)	97.2% (8.6)	524	97.6% (89.3–100%)	88.2 (24.7)	649
Q5 (M13–15)	99.2% (96.9–100%)	97.6% (8.0)	399	96.5% (89.2–100%)	87.3 (25.1)	487
Q6 (M16–18)	98.8% (96.1–100%)	96.9% (8.5)	238	96.3% (85.5–100%)	87 (26.2)	287
Q7 (M19–21)	98.8% (96.9–100%)	98.0% (4.5)	64	98.2% (91.0–100%)	90.3 (19.1)	96

a Unannounced pill counts and MEMS were planned for all 1,147 participants. For 46 participants, however, MEMS data were not expected because of enrollment shortly prior to the data analysis cut-off date and the lack of a subsequent clinic visit for uploading MEMS data. MEMS data were not available for eight (0.7%) of 1,101 participants with expected MEMS data owing to factors such as missing visits, device malfunction, or device loss. Similarly, for 70 participants, enrollment was too close to the data analysis cut-off date to expect a UPC following initiation of pill counting. Attempts at UPC were not successful for 36 (3.3%) of the remaining 1,077 participants.

Q, quarter; M, month.

### Factors associated with low (<80%) adherence

Tables 3 and 4 present the univariable and multivariable regression analyses for <80% adherence by UPC and MEMS, respectively. Incident pregnancy and reports of abuse (verbal, physical, and economic; assessed monthly) were of interest, but too rare to assess for potential associations with adherence. Factors independently associated with <80% UPC adherence on multivariable analysis (referencing the HIV-uninfected partner, unless otherwise stated) were report of no sexual activity (adjusted odds ratio [AOR] = 4.2; 95% CI 1.9–9.4) and sex with both the study partner and another partner (AOR = 3.0; 95% CI 1.5–5.9) within the previous month, younger age (AOR = 1.4; 95% CI 1.0–2.0; per decade), and heavy alcohol use (AOR = 2.8; 95% CI 1.4–5.5). Being in a formal polygamous marriage (i.e., not simply having more than one sexual partnership; AOR = 0.4; 95% CI 0.2–0.9) was associated with a lower likelihood of <80% UPC adherence. Similarly, factors independently associated with <80% MEMS adherence were report of no sex (AOR = 2.3; 95% CI 1.5–3.3) and sex with both the study partner and another partner (AOR = 1.6; 95% CI 1.1–2.4) in the previous month, and younger age (AOR = 1.7; 95% CI 1.3–2.1; per decade). Being in a polygamous relationship was also associated with a lower likelihood of <80% MEMS adherence (AOR = 0.6; 95% CI 0.4–1.0). Additional associations with <80% adherence seen only in the MEMS model were sex only with a partner other than the study partner (AOR = 2.3; 95% CI 1.3–3.8), and shorter time taking PrEP (AOR = 0.5; 95% CI 0.3–0.8) for 1–6 mo compared to more than 24 mo on PrEP. Heavy alcohol use in the HIV-uninfected partner was not a significant factor in the MEMS model. Testing for interactions between gender and sexual behavior suggested that women may have stronger associations with low adherence and having an outside partner compared with men (AOR for having an outside partner = 3.5 for women versus 0.8 for men by UPC, and AOR = 6.4 for women versus 1.6 for men by MEMS), but differences in associations by gender were not statistically significant (*p* = 0.15 and *p* = 0.25, respectively). No difference in the effect of polygamy was found by gender in either MEMS or UPC.

**Table 3:** Univariable and multivariable regressions of factors correlating with <80% unannounced pill count.

Factors	Prevalence or Mean (SD)	Quarters with <80% Adherence^a^	Univariable OR (95% CI)	*p*-Value*	Multivariable AOR (95% CI)	*p*-Value*
***HIV-uninfected partner, enrollment characteristics***						
Younger age (per decade)	35.7 (8.2)	32.7 (7.0)	1.7 (1.2–2.3)	**0.001**	1.4 (1.0–2.0)	**0.04**
Male	53%	53 (2.7%)	1.6 (1.0–2.7)	**0.05**	1.0 (0.5–1.7)	0.87
Randomized to active study drug (versus placebo)	65%	53 (2.2%)	1.1 (0.6–1.8)	0.83	—	—
Years of education≥6	52%	47 (2.4%)	1.2 (0.8–2.0)	0.39	—	**—**
***HIV-infected partner, enrollment characteristics***						
CD4 count:				0.28		
<350 cells/µl	25%	14 (1.5%)	0.6 (0.3–1.2)		—	—
350–500 cells/µl	29%	25 (2.3%)	0.9 (0.5–1.6)			
>500 cells/µl	46%	43 (2.5%)	reference			
***HIV-uninfected partner, time varying characteristics (in the past quarter)***						
Socio-economic status index	−0.01 (1.00)	0.31 (1.09)	1.2 (1.0–1.5)	**0.05**	1.2 (0.9–1.6)	0.21
Primary income from farming	60%	34 (1.5%)	0.5 (0.3–0.8)	**<0.01**	0.7 (0.3–1.3)	0.23
Heavy alcohol use	6%	10 (4.2%)	2.2 (1.1–4.5)	**0.03**	2.8 (1.4–5.5)	**0.004**
Depression	5%	2 (1.0%)	0.5 (0.1–1.9)	0.13	—	—
Travel time from home to clinic:				0.52		
<30 min	2%	1 (1.5%)	0.7 (0.4–1.3)			
30–59 min	10%	11 (2.9%)	1.2 (0.5–2.9)		—	**—**
1–2 h	35%	22 (1.7%)	0.6 (0.1–4.4)			
>2 h	53%	46 (2.3%)	reference			
Number of side effects	0.4 (0.6)	0.4 (0.6)	0.9 (0.6–1.4)	0.68	—	—
Sexual behavior in the previous month				**0.0004**		**0.01**
No sex	5%	10 (5.7%)	4.5 (2.1–9.4)		4.2 (1.9–9.4)	
Primary partner only, 100% condom use	55%	27 (1.3%)	reference		reference	
Primary partner only, <100% condom use	22%	20 (2.5%)	1.9 (1.0–3.4)		1.7 (0.9–3.1)	
Other partner only	2%	2 (2.2%)	1.7 (0.4–7.0)		1.5 (0.3–7.0)	
Other partner+primary partner	15%	21 (3.7%)	2.3 (1.2–4.3)		3.0 (1.5–5.9)	
Disclosure of partner's HIV status to anyone	68%	57 (2.3%)	1.2 (0.6–2.1)	0.64	—	**—**
Belief in PrEP: HIV medicines prevent HIV	25%	18 (1.9%)	0.9 (0.5–1.6)	0.67		
PrEP use before sex prevents HIV	15%	8 (1.5%)	0.7 (0.3–1.5)	0.25	—	—
The study pill makes sex safe	19%	12 (1.7%)	0.8 (0.4–1.5)	0.43		
Months on PrEP				0.08		0.45
1–6 mo	17%	15 (2.4%)	1.9 (0.8–4.5)		1.3 (0.5–3.3)	
7–12 mo	22%	26 (3.2%)	2.5 (1.1–5.5)		1.8 (0.8–4.0)	
13–18 mo	22%	20 (2.5%)	1.9 (0.9–4.3)		1.4 (0.6–3.3)	
19–24 mo	21%	10 (1.3%)	1.0 (0.4–2.4)		0.9 (0.4–2.2)	
25+ mo^b^	19%	9 (1.3%)	reference		reference	
***HIV-infected partner, time-varying characteristics (in the past quarter)***						
CD4 count:				0.30		
<200 cells/µl	5%	4 (2.1%)	0.9 (0.3–3.1)			
200–349 cells/µl	24%	14 (1.6%)	0.7 (0.4–1.2)		—	—
>350 cells/µl	71%	64 (2.4%)	reference			
On ART	16%	7 (1.2%)	0.5 (0.2–1.3)	0.15	—	**—**
***Partnership, enrollment characteristics***						
Not living together	2%	2 (2.9%)	1.3 (0.3–5.7)	0.70	—	—
No children with partner	20%	20 (2.6%)	0.6 (0.2–1.8)	0.34	—	**—**
Polygamous marriage	23%	10 (1.1%)	0.4 (0.2–0.9)	**0.02**	0.4 (0.2–0.9)	**0.03**

Less than 80% adherence was seen among 71 participants in 2.3% of study quarters. UPC data available were available for 3,766 of 4,361 (86.4%) of study quarters.

a *n* (row %) or mean (SD).

b UPC data available were available for 3,766 of 4,361 (86.4%) of study quarters.

* Bold indicates *p*<0.05.

**Table 4:** Univariable and multivariable regressions of factors correlating with <80% electronic monitoring adherence.

Factors	Prevalence or Mean (SD)	Quarters with <80% Adherence^a^	Univariable OR (95% CI)	*p*-Value*	Multivariable AOR (95% CI)	*p*-Value*
***HIV-uninfected partner, enrollment characteristics***						
Younger age (per decade)	35.7 (8.2)	32.1 (7.3%)	2.0 (1.6–2.5)	**<0.001**	1.7 (1.3–2.1)	**0.01**
Male	53%	423 (18.1%)	2.0 (1.5–2.7)	**<0.001**	1.3 (0.9–1.9)	0.16
Randomized to active study drug (versus placebo)	65%	417 (14.8%)	1.1 (0.8–1.5)	0.50	—	—
Years of education ≥6	52%	416 (19.1%)	2.0 (1.5–2.7)	**<0.001**	1.0 (0.7–1.4)	0.96
***HIV-infected partner, enrollment characteristics***						
CD4 count:				0.03		
<350 cells/µl	25%	113 (10.8%)	0.7 (0.4–0.9)		—	—
350–500 cells/µl	29%	183 (14.2%)	0.8 (0.6–1.2)			
>500 cells/µl	46%	329 (16.3%)	reference			
***HIV-uninfected partner, time varying characteristics (in the past quarter)***						
Socio-economic status index	−0.01 (1.00)	0.57 (1.15)	1.6 (1.4–1.8)	**<0.001**	1.1 (0.9–1.3)	0.47
Primary income from farming	60%	223 (8.5%)	0.3 (0.2–0.4)	**<0.001**	0.8 (0.5–1.2)	0.22
Heavy alcohol use	6%	40 (14.5%)	1.0 (0.6–1.7)	0.91	—	**—**
Depression	5%	36 (15.7%)	1.1 (0.6–2.1)	0.67	—	—
Travel time from home to clinic:				**0.01**		0.25
<30 min	2%	8 (10.7%)	0.9 (0.3–2.8)		0.6 (0.2–1.9)	
30–59 min	10%	96 (22.4%)	2.2 (1.4–3.3)		1.3 (0.8–2.1)	
1–2 h	35%	239 (16.0%)	1.4 (1.1–1.9)		0.9 (0.6–1.2)	
>2 h	53%	277 (11.8%)	reference		reference	
Number of side effects	0.4 (0.6)	0.4 (0.6)	0.9 (0.7–1.1)	0.20	—	—
Sexual behavior in the past month				**<0.001**		**<0.001**
No sex	5%	64 (25.6%)	2.7 (1.9–3.8)		2.3 (1.5.3.3)	
Primary partner only, 100% condom use	55%	267 (11.9%)	reference		reference	
Primary partner only, <100% condom use	22%	123 (13.0%)	1.2 (0.8–1.6)		1.1 (0.8–1.6)	
Other partner only	2%	45 (39.1%)	4.8 (2.9–7.9)		2.3 (1.3–3.8)	
Other partner+primary partner	15%	119 (17.7%)	1.7 (1.2–2.4)		1.6 (1.1–2.4)	
Disclosure of partner's HIV status to anyone	68%	346 (11.6%)	0.5 (0.4–0.7)	**<0.001**	1.0 (0.8–1.4)	0.79
Belief in PrEP: HIV medicines prevent HIV	25%	121 (10.7%)	0.7 (0.5–0.9)	**0.01**	1.1 (0.7–1.6)	0.76
PrEP use before sex prevents HIV	15%	109 (13.5%)	0.9 (0.6–1.4)	0.73	—	—
The study pill makes sex safe	19%	70 (8.2%)	0.5 (0.4–0.6)	**<0.001**	0.7 (0.5–1.1)	0.13
Months on PrEP				**0.01**		**<0.001**
1–6 mo	17%	94 (13.8%)	1.2 (0.8–1.8)		0.5 (0.3–0.8)	
7–12 mo	22%	172 (17.8%)	1.6 (1.2–2.3)		0.9 (0.6–1.4)	
13–18 mo	22%	141 (14.6%)	1.3 (0.9–1.8)		0.8 (0.6–1.2)	
19–24 mo	21%	114 (12.8%)	1.1 (0.8–1.5)		1.0 (0.7–1.4)	
25+ mo	19%	99 (11.7%)	reference		reference	
***HIV-infected partner, time-varying characteristics (in the past quarter)***						
CD4 count:				0.09		0.82
<200 cells/µl	5%	24 (16.1%)	1.1 (0.6–1.9)		1.1 (0.6–1.9)	
200–350 cells/µl	24%	120 (11.5%)	0.7 (0.5–1.0)		1.1 (0.8–1.6)	
>350 cells/µl	71%	481 (15.2%)	reference		reference	
On ART	16%	68 (9.9%)	0.6 (0.4–0.9)	**0.03**	0.8 (0.5–1.2)	0.21
***Partnership, enrollment characteristics***						
Not living together	2%	8 (9.8%)	0.6 (0.2–2.0)	0.43	—	—
No children with partner	20%	207 (23.2%)	2.3 (1.4–3.9)	**0.001**	1.0 (0.6–1.7)	0.92
Polygamous marriage	23%	85 (8.4%)	0.6 (0.4–0.8)	**<0.001**	0.6 (0.4–1.0)	**0.03**

Less than 80% adherence was seen among 282 participants in 14.4% of study quarters. MEMS data were available for 4,357 of 4,463 (97.2%) study quarters.

a *n* (row %) or mean (SD).

* Bold indicates *p*<0.05.

### Adherence intervention

At the time of the analysis cut-off date (July 2011), a total of 124 participants (10.8%) were observed to have <80% UPC adherence. Of these, 13 triggered just prior to the cut-off date, and 103 (92.8% of the 111 remaining) received at least one intervention session. The intervention was well received with only one participant declining to participate. A UPC following the intervention was available for 66 participants as of the cut-off date. UPC adherence improved to ≥80% in 61 participants (92%), and 54 (82%) remained at ≥80% for the remainder of UPCs performed.

## Discussion

In this substudy of adherence nested within a randomized clinical trial of PrEP among African HIV serodiscordant couples, where participants received a combination of both adherence monitoring and intensive counseling when adherence dropped below 80%, adherence to PrEP was high by two objective, validated measures and efficacy of PrEP was 100% (95% CI 83.7%–100%). Because high adherence is a prerequisite for measured efficacy to approximate biologic efficacy [25], these results provide confidence in the high efficacy estimate for protection against HIV found in the larger Partners PrEP Study. The lack of seroconversions among the adherence study participants randomized to PrEP provides further support that PrEP is highly efficacious against HIV acquisition among highly adherent PrEP users.

Despite the overall high levels of adherence, adherence <80% was observed at some point during a quarterly follow-up interval in as many as 25.8% of participants over an average of 11.3 mo of follow-up. Sexual behavior was closely associated with PrEP adherence. Those participants who reported not having sex were less likely to adhere to PrEP during that study quarter than those reporting sex, presumably because they did not perceive themselves to be at risk during periods of no sexual activity. Similarly, participants who reported having sex with another partner (with or without having sex with the primary partner) may perceive themselves to be at lower risk, especially if their outside partner is known to be HIV-uninfected. Additionally, partners within a formal polygamous marriage were more likely to adhere, suggesting a desire to reduce the risk of HIV acquisition within multiple stable and committed partnerships.

Younger age and heavy alcohol use in the HIV-uninfected partner were associated with a greater likelihood of low PrEP adherence; these factors are well established as being associated with lower adherence to antiretroviral treatment in HIV-infected people [26],[27]. The finding of higher adherence in the first 6 mo of use may reflect initial enthusiasm for a novel prevention method that may be challenging to sustain over time. Waning adherence patterns have been seen with daily oral contraceptive pills [28] and strategies to maintain good adherence over time may be needed.

Adherence counseling, both in the routine sessions and in the adherence intervention, may have played a role in the high adherence seen in this study. Adherence for most participants did increase after the intervention, although the study was not designed to assess the efficacy of the intervention. Implementation challenges, however, may influence the extent of counseling to be provided as PrEP becomes available in demonstration projects and ultimately clinical care. Further research should focus on identifying key adherence counseling messages, standardized approaches for providing appropriate counseling within the “real world” context, and the cost-effectiveness of adherence interventions. Identifying appropriate counseling approaches will be critical to ensure the behavioral success of this biological agent for HIV prevention.

Adherence is difficult to compare among the PrEP clinical trials that lack comparable measures of adherence behavior. That said, our data and previously reported data suggest that the degree of HIV protection is highly correlated with adherence. The highest levels of PrEP efficacy have been reported for the HIV serodiscordant couples in the Partners PrEP Study with 75% protection from FTC/TDF and 67% from TDF [3]. In the TDF-2 study, FTC/TDF conferred 62% protection in young, heterosexual men and women from Botswana who were recruited regardless of their partner's serostatus [5], and the iPrEX study found 44% protection against HIV infection from FTC/TDF among men who have sex with men [4]. The degree of protection and corresponding adherence may be the highest in the Partners PrEP Study because the HIV-uninfected partner taking PrEP received a higher level of adherence support from his or her HIV-infected partner and both partners recognized the risk of HIV transmission [29]. Given that up to 20% of couples in sub-Saharan Africa are serodiscordant [30], this population may be an ideal target for initial PrEP implementation strategies. Counseling of the couple, or another identified support partner for individuals taking PrEP outside of a partnership, may be a key factor for the success of PrEP beyond clinical trials. It is important to note, however, that fewer than 40% of individuals living with HIV know their serostatus [31]. Further efforts will therefore be needed to scale up counseling and testing services to identify serodiscordant couples.

The strengths of this study include the use of two objective behavioral adherence measures; a large sample size; a robust set of socio-demographic, biological, and behavioral factors potentially associated with adherence; and the availability of HIV seroconversion data within a clinical trial. This study also has important limitations. First, no adherence measure is perfect. Although UPC and MEMS are significantly correlated and both indicate high adherence, UPC is consistently somewhat higher than MEMS. This relationship suggests systematic biases, which have been similarly reported in the literature [32]. We believe this difference primarily reflects the removal of multiple doses from the MEMS pill bottle during a single opening, as may occur when an individual travels without their pill bottle (often due to inconvenience and/or stigma) [33]. Pills lost in between pill counts may also contribute to misclassification. Pill sharing could also contribute to misclassification; however, there was no self-reported pill sharing in this substudy. While social desirability may cause such self-report to be an underestimate, the high efficacy reported here and in the clinical trial would be hard to achieve with widespread pill sharing (see Baeten et al., supplementary materials) [3]. True adherence likely lies somewhere in between the two measurements. Second, due to the small numbers of participants with low adherence as measured by UPC, the power to identify factors associated with that measure of adherence was limited. Factors such as abuse may also be underreported and therefore difficult to identify. Third, this substudy was conducted within a blinded randomized controlled trial and recruitment was performed without regard to study arm. Although there were some differences in the baseline participant characteristics between the adherence substudy and the clinical trial, these differences were relatively minor, especially when data are restricted to those sites at which the substudy took place, and no meaningful differences were seen across the study arms. It is, however, possible that these differences influenced the efficacy estimate. Finally, the 80% threshold may or may not reflect the optimal level of adherence for protection against HIV acquisition. This study cannot assess whether non-adherence correlated with HIV infection because no individuals in the treatment groups became infected.

Identifying participants with <80% adherence for intensification of adherence counseling may have played an important role in achieving high efficacy in this adherence study. However, timely identification of adherence problems in general is a challenge even within clinical trials. Incomplete adherence is typically detected weeks to months after it occurs, which in the case of PrEP may result in seroconversion. Real-time adherence monitoring has recently been shown to be feasible within developing settings [34]. If affordable, such monitoring could be used to identify people taking PrEP for targeted, enhanced adherence support.

In summary, we found both high levels of adherence and a high degree of protection against HIV infection in a substudy within a clinical trial of oral PrEP using two objective and validated measures of adherence. These data provide further support that PrEP is highly efficacious at preventing HIV acquisition when it is taken. Our data also suggest that future development of risk reduction strategies and adherence interventions in the implementation setting should address sexual behavior, risk perception, and heavy alcohol use, especially for young PrEP takers and prolonged PrEP use. Proper support and assessment of adherence will be critical for determining efficacy of PrEP outside of clinical trials. This data will be important for guiding ethical decisions about resource allocation for both prevention and treatment of HIV.

## Abbreviations

AOR: adjusted odds ratio
FTC/TDF: emtricitabine/tenofovir
IQR: interquartile range
IRR: incidence rate ratio
MEMS: medication event monitoring system
PrEP: pre-exposure prophylaxis
TDF: tenofovir
UPC: unannounced home-based pill counts

## References

[1] UNAIDS/WHO (2010) AIDS epidemic update. Available: http://www.unaids.org/globalreport. Accessed: 15 October 2012.

[2] Padian NS, McCoy SI, Karim SS, Hasen N, Kim J, et al (2011) HIV prevention transformed: the new prevention research agenda. Lancet 378: 269–278. 21763938 10.1016/S0140-6736(11)60877-5 PMC3606928

[3] Baeten J, Donnell D, Ndase P, Mugo NR, Campbell JD, et al (2012) Antiretroviral prophylaxis for HIV prevention in heterosexual men and women. N Engl J Med 367: 399–410. 22784037 10.1056/NEJMoa1108524 PMC3770474

[4] Grant RM, Lama JR, Anderson PL, McMahan V, Liu AY, et al (2010) Preexposure chemoprophylaxis for HIV prevention in men who have sex with men. N Engl J Med 363: 2587–2599. 21091279 10.1056/NEJMoa1011205 PMC3079639

[5] Thigpen MC, Kebaabetswe PM, Paxton LA, Smith DK, Rose CE, et al (2012) Antiretroviral preexposure prophylaxis for heterosexual HIV transmission in Botswana. N Engl J Med 367: 423–434. 22784038 10.1056/NEJMoa1110711

[6] Microbicide Trials Network (MTN) (2011) Statement on decision to discontinue use of oral tenofovir tablets in VOICE, a major HIV prevention study in women. Available: http://www.mtnstopshiv.org/node/3619. Accessed 15 October 2012.

[7] Van Damme L, Corneli A, Ahmed K, Agot K, Lombaard J, et al (2012) Preexposure prophylaxis for HIV infection among African women. N Engl J Med 367: 411–422. 22784040 10.1056/NEJMoa1202614 PMC3687217

[8] Williams A, Friedland G (1997) Adherence, compliance, and HAART. AIDS Clin Care 9: 51–58, 51-54, 58. 11364415

[9] Kashuba ADM, Patterson KB, Dumond JB, Cohen MS (2012) Pre-exposure prophylaxis for HIV prevention: how to predict success. Lancet 6736: 61852–61857. 10.1016/S0140-6736(11)61852-7 PMC3652584 22153566

[10] van der Straten A, Van Damme L, Haberer JE, Bangsberg DR (2012) Unraveling the divergent results of pre-exposure prophylaxis trials for HIV prevention. AIDS 26: F13–F19. 22333749 10.1097/QAD.0b013e3283522272

[11] Marrazzo J, Ramjee G, Nair G, Palanee T, Mkhize B, et al. (2013) Pre-exposure prophylaxis for HIV in women: daily oral tenofovir, oral tenofovir/emtricitabine, or vaginal tenofovir gel in the VOICE study (MTN 003). 20th Conference on Retroviruses and Opportunistic Infections; Atlanta, 3–6 March 2013; paper number 26LB.

[12] Anderson PL, Glidden DV, Liu A, Buchbinder S, Lama JR, et al (2012) Emtricitabine- tenofovir concentrations and pre-exposure prophylaxis efficacy in men who have sex with men. Sci Transl Med 4: 151ra25. 10.1126/scitranslmed.3004006 PMC3721979 22972843

[13] Podsadecki TJ, Vrijens BC, Tousset EP, Rode RA, Hanna GJ (2008) “White coat compliance” limits the reliability of therapeutic drug monitoring in HIV-1-infected patients. HIV Clin Trials 9: 238–246. 18753118 10.1310/hct0904-238

[14] Simoni JM, Kurth AE, Pearson CR, Pantalone DW, Merrill JO, et al (2006) Self-report measures of antiretroviral therapy adherence: A review with recommendations for HIV research and clinical management. AIDS Behav 10: 227–245. 16783535 10.1007/s10461-006-9078-6 PMC4083461

[15] Hugen PW, Langebeek N, Burger DM, Zomer B, van Leusen R, et al (2002) Assessment of adherence to HIV protease inhibitors: comparison and combination of various methods, including MEMS (electronic monitoring), patient and nurse report, and therapeutic drug monitoring. J Acquir Immune Defic Syndr 30: 324–334. 12131570 10.1097/00126334-200207010-00009

[16] Liechty CA, Alexander CS, Harrigan PR, Guzman JD, Charlebois ED, et al (2004) Are untimed antiretroviral drug levels useful predictors of adherence behavior? AIDS 18: 127–129. 15090840 10.1097/00002030-200401020-00017

[17] Bangsberg DR, Hecht FM, Charlebois ED, Zolopa AR, Holodniy M, et al (2000) Adherence to protease inhibitors, HIV-1 viral load, and development of drug resistance in an indigent population. AIDS 14: 357–366. 10770537 10.1097/00002030-200003100-00008

[18] Oyugi JH, Byakika-Tusiime J, Charlebois ED, Kityo C, Mugerwa R, et al (2004) Multiple validated measures of adherence indicate high levels of adherence to generic HIV antiretroviral therapy in a resource-limited setting. J Acquir Immune Defic Syndr 36: 1100–1102. 15247564 10.1097/00126334-200408150-00014

[19] Psaros C (2011) An adherence intervention to support HIV pre-exposure prophylaxis (PrEP) adherence in serodiscordant couples in Uganda. 6th Annual International Conference on HIV Treatment and Prevention Adherence; Miami, 22–24 May 22–24; number 70065.

[20] Garcia-Lerma JG, Otten RA, Qari SH, Jackson E, Cong ME, et al (2008) Prevention of rectal SHIV transmission in macaques by daily or intermittent prophylaxis with emtricitabine and tenofovir. PLoS Med 5: e28 doi:10.1371/journal.pmed.0050028 18254653 10.1371/journal.pmed.0050028 PMC2225435

[21] Abdool Karim Q, Abdool Karim SS, Frohlich JA, Grobler AC, Baxter C, et al (2010) Effectiveness and safety of tenofovir gel, an antiretroviral microbicide, for the prevention of HIV infection in women. Science 329: 1168–1174. 20643915 10.1126/science.1193748 PMC3001187

[22] Filmer D, Pritchett LH (2001) Estimating wealth effects without expenditure data—or tears: an application to educational enrollments in states of India. Demography 38: 115–132. 11227840 10.1353/dem.2001.0003

[23] Cherpitel CJ, Ye Y, Bond J, Borges G, Cremonte M, et al (2005) Cross-national performance of the RAPS4/RAPS4-QF for tolerance and heavy drinking: data from 13 countries. J Stud Alcohol 66: 428–432. 16047534 10.15288/jsa.2005.66.428

[24] Bolton P, Wilk CM, Ndogoni L (2004) Assessment of depression prevalence in rural Uganda using symptom and function criteria. Soc Psychiatry Psychiatr Epidemiol 39: 442–447. 15205728 10.1007/s00127-004-0763-3

[25] Weiss HA, Wasserheit JN, Barnabas RV, Hayes RJ, Abu-Raddad LJ (2008) Persisting with prevention: the importance of adherence for HIV prevention. Emerg Themes Epidemiol 5: 8. 18620578 10.1186/1742-7622-5-8 PMC2507711

[26] Hahn JA, Woolf-King SE, Muyindike W (2011) Adding fuel to the fire: alcohol's effect on the HIV epidemic in Sub-Saharan Africa. Curr HIV/AIDS Rep 8: 172–180. 21713433 10.1007/s11904-011-0088-2

[27] Smith RM, Carrico AW, Montandon M, Kwena Z, Bailey R, et al (2011) Attitudes and beliefs about anti-retroviral therapy are associated with high risk sexual behaviors among the general population of Kisumu, Kenya. AIDS Care 23: 1668–1675. 22050441 10.1080/09540121.2011.579947 PMC3682819

[28] Pittman ME, Secura GM, Allsworth JE, Homco JB, Madden T, et al (2011) Understanding prescription adherence: pharmacy claims data from the Contraceptive CHOICE Project. Contraception 83: 340–345. 21397092 10.1016/j.contraception.2010.08.003 PMC3058146

[29] Ware NC, Wyatt MA, Haberer JE, Baeten JM, Kintu A, et al (2012) What's love got to do with it? Explaining adherence to oral antiretroviral pre-exposure prophylaxis for HIV-serodiscordant couples. J Acquir Immune Defic Syndr 59: 463–468. 22267018 10.1097/QAI.0b013e31824a060b PMC3826169

[30] Eyawo O, de Walque D, Ford N, Gakii G, Lester RT, et al (2010) HIV status in discordant couples in sub-Saharan Africa: a systematic review and meta-analysis. Lancet Infect Dis 10: 770–777. 20926347 10.1016/S1473-3099(10)70189-4

[31] UNAIDS (2010) Getting to zero: 2011–2015 strategy. Available: https://www.unaids.org/en/media/unaids/contentassets/documents/unaidspublication/2010/JC2034_UNAIDS_Strategy_en.pdf. Accessed 28 January 2013.

[32] Bangsberg DR, Hecht FM, Charlebois ED, Chesney M, Moss A (2001) Comparing Objective measures of adherence to HIV antiretroviral therapy: electronic medication monitors and unannounced pill counts. AIDS Behav 5: 275–281.

[33] Safren SA, Kumarasamy N, Hosseinipour M, Harwood MM, Hoffman I, et al (2006) Perceptions about the acceptability of assessments of HIV medication adherence in Lilongwe, Malawi and Chennai, India. AIDS Behav 10: 443–450. 16604297 10.1007/s10461-006-9094-6

[34] Haberer JE, Kahane J, Kigozi I, Emenyonu N, Hunt P, et al (2010) Real-time adherence monitoring for HIV antiretroviral therapy. AIDS Behav 14: 1340–1346. 20809380 10.1007/s10461-010-9799-4 PMC2974938

